# Extensive subcutaneous emphysema with pneumothorax in COVID-19

**DOI:** 10.11604/pamj.2023.44.84.36292

**Published:** 2023-02-10

**Authors:** Mohammed Ali, Salim Surani

**Affiliations:** 1 Department of Pulmonary Medicine, Corpus Christi Medical Center, Texas, United States,; 2 Department of Pulmonary, Critical Care and Sleep Medicine, Texas A and M University Health Science Center, Texas, United States

**Keywords:** Spontaneous pneumothorax, pneumothorax, COVID-19, subcutaneous emphysema

## Image in medicine

Subcutaneous emphysema is the infiltration of air in the subcutaneous tissues. There are multiple causes of subcutaneous emphysema that can occur, for example, due to surgery, infection, barotrauma, trauma, or spontaneous. In a patient with respiratory failure on mechanical ventilation especially in severe Acute respiratory distress syndrome (ARDS), positive pressure ventilation may lead to subcutaneous emphysema. During positive pressure ventilation, small mucosal injury in the trachea or the bronchi leads air to escape out of the airways. The air then travels along the endovascular bundle into the mediastinum and then follows the facial planes into the neck and subcutaneous tissue of the chest. Positive pressure ventilation may also lead to a pneumothorax due to barotrauma or volutrauma. The subsequent injury to the parietal pleura causes air to leak into the subcutaneous tissue of the chest. This is a 71-year-old female with a history of type 2 diabetes, hypertension, and obesity, who was admitted for acute hypoxic respiratory failure secondary to COVID-19. In the emergency room (ER) her oxygen saturation was 75% on room air, and she was started on supplemental oxygen. Laboratory workup revealed a normal complete blood, count and complete metabolic panel, c-reactive protein (CRP) was elevated at 13.5 mg/dL and COVID-19 polymerase chain reaction (PCR) was positive. Initial chest X-ray was also normal with very mild right lower lobe infiltrate. On hospital day 10, she was intubated for worsening respiratory failure. Seven (7) days post-intubation, chest X-ray (A) revealed significant subcutaneous emphysema (blue arrows). A computed tomography (CT) scan of the chest (B) was obtained which revealed a left pneumothorax (red arrow) with significant

**Figure 1:**
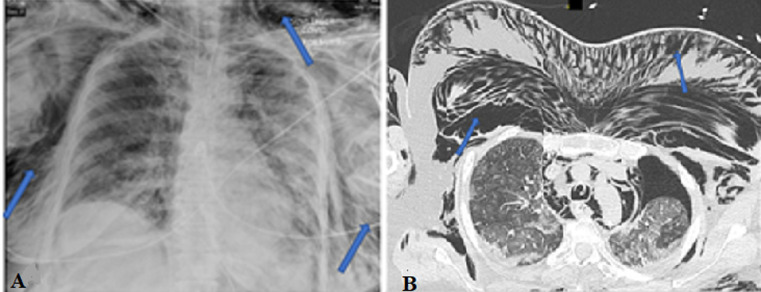
A) the X-ray chest AP view showing significant subcutaneous emphysema; B) the CT scan of chest with the blue arrows showing extensive subcutaneous emphysema

